# On the Same Page: Building Best Practices of Peer Coaching for Medical Educators Using Nominal Group Technique

**DOI:** 10.15694/mep.2019.000095.1

**Published:** 2019-04-25

**Authors:** Adriane Bell, Holly Meyer, Lauren Maggio

**Affiliations:** 1 Uniformed Services University of the Health Sciences

**Keywords:** peer coach, faculty development, observation, feedback, collegial exchange, nominal group technique

## Abstract

This article was migrated. The article was marked as recommended.

**Introduction**: Peer coaching is a faculty development approach that improves teaching practice. Elements include peer observation of teaching, feedback, and collegial exchange. Peer coaching supports reflection on teaching, cultivates workplace learning, and fosters learning cultures. Yet, limited resources are available to guide faculty developers in designing and implementing peer coaching initiatives. This gap may lead to initiatives that fail to optimize teaching effectiveness.

**Methods:** The authors convened a focus group of seven participant experts, via video-teleconference, to arrive at consensus on best practices of peer coaching for medical educators. The focus group utilized Nominal Group Technique, a consensus building methodology. Process steps included an introduction, silent idea generation, idea sharing, group discussion, and voting. Consensus was reached with over 50% agreement. Data were qualitatively analyzed using inductive content analysis, and quotes were extracted to support the identification of best practices.

**Results:** Seventeen best practices were identified. All participant experts recommended a framework for the peer observation process including a pre-observation meeting and post-observation debrief. The participant experts stressed the importance of confidentiality and behaviorally-based feedback. To promote collegial exchange, most agreed peer coaching should be a formative process conducted in an environment that is safe and nonthreatening. Finally, peer coaching should be supported at multiple levels within an organization.

**Conclusion:** Expert consensus generated 17 best practices of peer coaching for medical educators that optimize teaching effectiveness. The results provide a practical resource for faculty developers. Future researchers should explore common pitfalls and barriers to the implementation of peer coaching initiatives from the perspectives of academic leadership, peer coaches, and observed educators.

## Introduction

Peer coaching is a faculty development approach in which educators collegially work together to improve teaching practice ( Huston and Weaver, 2008). Research demonstrates that peer coaching promotes reflection and collaboration on teaching ( Martin and Double, 1998; Hammersley, Fletcher and Orsmond, 2005; Finn *et al.*, 2011; Pierce Jr, Rendón and Rao, 2018). Moreover, peer coaching cultivates workplace learning and fosters positive learning cultures ( Billett, 2001; Huston and Weaver, 2008; Devine, Meyers and Houssemand, 2013). Despite these benefits, there are few resources to guide faculty developers in peer coaching initiative design and implementation for medical educators ( Siddiqui, Jonas-Dwyer and Carr, 2007; Steinert *et al.*, 2016; Newman, Roberts and Frankl, 2018). This gap may lead to initiatives that fail to improve teaching practice.

Direct observation of teaching with associated feedback and collegial exchange are common components of peer coaching ( Huston and Weaver, 2008; Boillat and Elizov, 2014). Direct observation may occur in classroom or clinical settings (e.g., outpatient clinic, inpatient ward, operating room) ( Sekerka and Chao, 2003; Newman *et al.*, 2009; Mai and Baker, 2017; Pierce Jr, Rendón and Rao, 2018). Peer coaching can involve an expert-to-novice, instructional coaching style or a peer-to-peer, reciprocal coaching style ( Huston and Weaver, 2008; Devine, Meyers and Houssemand, 2013). By encouraging reflection on teaching and dialogue surrounding instructional strategies and teaching challenges, peer coaching promotes collegial exchange ( Newman, Roberts and Frankl, 2018; Pierce Jr, Rendón and Rao, 2018). Through this process, educators gain new perspectives on teaching and attain motivation to change ( Cordingley and Buckler, 2012).

Peer coaching offers a host of benefits for participants. For example, participating in peer coaching has been linked to improvements in teacher satisfaction, increases in self-efficacy, and promotion of skill transfer to the teaching environment ( Regan-Smith, Hirschmann and Iobst, 2007; O’Keefe *et al.*, 2009; Finn *et al.*, 2011; Pattison *et al.*, 2012; Garcia *et al.*, 2017; Pierce Jr, Rendón and Rao, 2018). Additionally, studies indicate a learning benefit to the peer coach ( Finn *et al.*, 2011; Pierce Jr, Rendón and Rao, 2018). Specific to medical education, peer coaching aligns with suggestions to conduct faculty development activities in the workplace and to create opportunities to promote communities of practice. Both of which have been shown to be beneficial ( O’Sullivan and Irby, 2011; Steinert *et al.*, 2016).As a faculty development approach, peer coaching may also include consultation on teaching practices, such as curriculum design and assessment, and/or it may be used to support faculty roles in leadership development and research ( Boillat and Elizov, 2014).

Despite these benefits, there are limited resources to assist faculty developers in designing and implementing peer coaching initiatives in medical education. For example, published literature often describes individual peer coaching initiatives and outcome measures, but the findings lack generalizability ( Regan-Smith, Hirschmann and Iobst, 2007; O’Keefe *et al.*, 2009; Finn *et al.*, 2011; Pattison *et al.*, 2012; Garcia *et al.*, 2017; Pierce Jr, Rendón and Rao, 2018). There are two studies evaluating physician perceptions of peer observation prior to implementing an initiative, however, researchers have not conducted follow-up studies to assess whether participants’ perceptions changed and why. From a practical standpoint, two “Twelve Tips” articles have been published that provide advice for individual components of peer coaching including peer observation of teaching and providing feedback to peers ( Siddiqui, Jonas-Dwyer and Carr, 2007; Newman, Roberts and Frankl, 2018). While valuable, these articles did not examine peer coaching initiatives holistically or draw on multiple expert opinions.

The lack of consensus on peer coaching processes and characteristics that optimize teaching effectiveness may result in recreating initiatives that prove to be ineffective. Our study aims to answer: What are the best practices of peer coaching that optimize teaching effectiveness for medical educators? By answering this question, we seek to provide faculty developers with a practical resource to build effective initiatives, collaborate, and identify areas for future research.

## Methods

We convened a focus group guided by a constructivist paradigm to determine best practices of peer coaching for medical educators. The Uniformed Services University of the Health Sciences (USU) institutional review board declared this study exempt from further review (Protocol Number: T0839444).

### Methodology:

To structure the focus group, we used Nominal Group Technique (NGT), a consensus building methodology. We selected NGT because it promotes equal representation among group members and creates an environment that fosters collaboration ( Harvey and Holmes, 2012). Steps in the NGT process include an introduction and explanation of the purpose of the focus group, silent idea generation, sharing ideas, group discussion, and voting and/or ranking of ideas ( Potter, Gordon and Hamer, 2004). Upon conclusion of the voting, consensus results are immediately available to share with the group ( Potter, Gordon and Hamer, 2004).

### Sample:

AB invited 45 North American medical school faculty developers with expertise in developing and/or leading peer coaching initiatives to participate. This sample was based on a website review that identified all peer coaching initiatives in North America with a web presence. Participant experts’ names and contact information were obtained from each school’s website, and they were contacted via email. Twelve faculty developers acknowledged an interest in participating, and of those, seven were available to participate in our focus group at the scheduled date and time.

### Process:

We conducted a pilot NGT to test our online platform and study materials with faculty and health professions education students at USU. Feedback from the pilot was used to refine our focus group questions and voting methods. Throughout the study, we used the video-teleconference software Adobe Connect (San Jose, CA).

**Figure 1.**
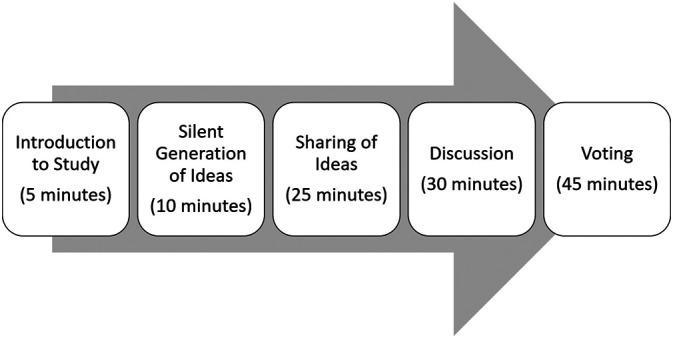
Steps of the Nominal Group Technique

We held the focus group on 10 May 2018 over a 2-hour period. AB led the meeting and was assisted in-person by HM and LM. Throughout the process, participant experts communicated via video, audio, and chat functions within Adobe Connect. To orient participant experts prior to our study, we provided a handout and made a brief presentation that reviewed our research aims and NGT methodology ( Figure 1).

**Figure 2.**
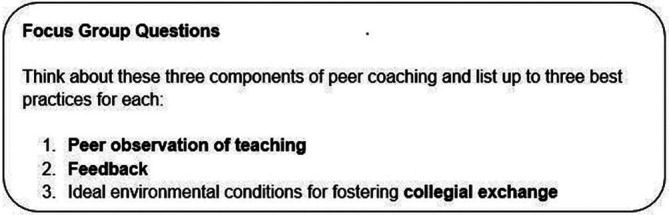
Focus Group Questions

Participants were presented with our definition of a best practice: 1) it is a standard component of a peer coaching initiative 2) that optimizes teaching effectiveness. Then, participant experts were asked a series of focus group questions that we derived from the literature and our website review ( Figure 2) ( Joyce and Showers, 1982; Huston and Weaver, 2008; Boillat and Elizov, 2014). We considered collegial exchange synonymous with collaborative reflection ( Martin and Double, 1998). It involves a trusting collegial relationship where peer coaching partners reflect together on challenging teaching situations and collectively develop strategies to overcome them ( Showers, 1984; Eisen, 2001; Boillat and Elizov, 2014).

During silent idea generation, participant experts individually wrote down answers to the focus group questions. Then, they shared key ideas (individual answers to focus group questions) with each other in a round robin format. Key ideas were categorized under peer observation of teaching, feedback or collegial exchange and displayed in Google Sheets (Mountain View, CA) for all participant experts to view in real-time. Next, they discussed key ideas within each category with the intention to consolidate similar ideas and to add new ideas if necessary.

**Figure 3.**
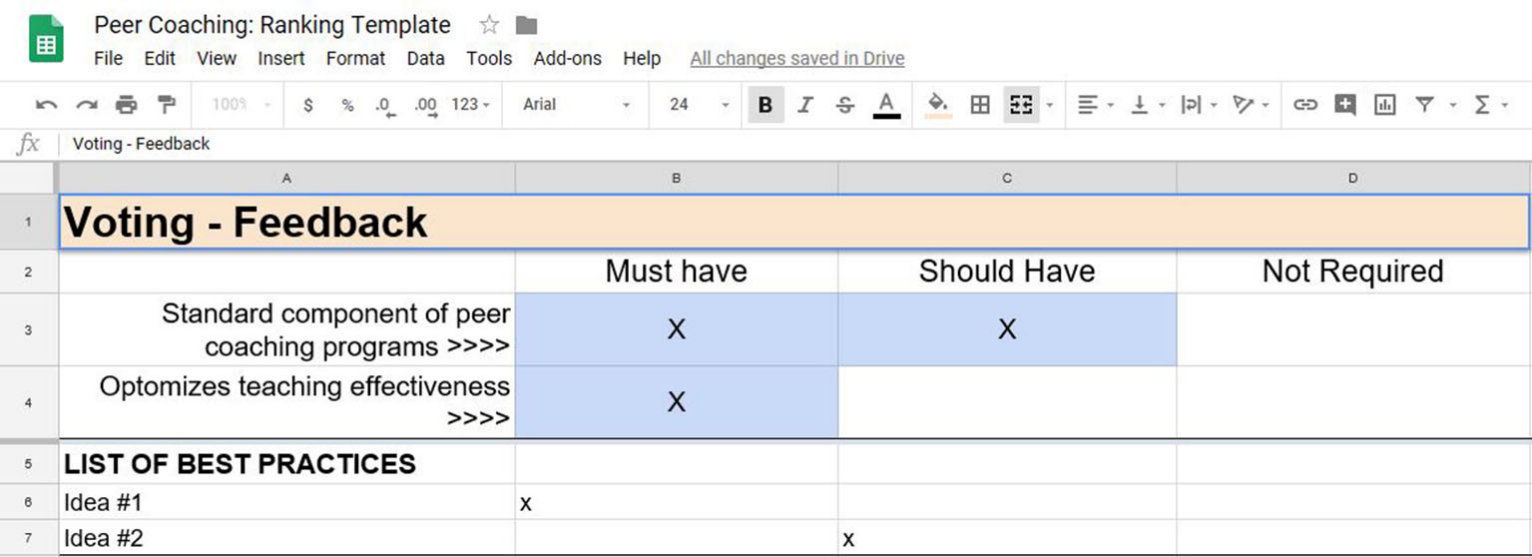
Example of Voting Spreadsheet

Finally, participant experts voted to determine which key ideas qualified as best practices. Consensus was considered >50% agreement on a key idea. Voting options included Must Have (Best Practice), Should Have, and Not Required. Figure 3 provides an example of our voting spreadsheet. Voting occurred anonymously using the Adobe Connect voting feature. If a vote resulted in a tie, a re-vote was cast once all key ideas were voted on within a category. Voting results were shared with the group in real-time.

### Data processing and analysis:

We applied a qualitative approach to data processing and analysis. Following the focus group, AB de-identified the data, transcribed the audio of the focus group and combined it with the video-teleconference chat log to further contextualize participant experts’ responses. Within each category, the list of key ideas was organized based on voting results (Best Practice, Should Have, Not Required). These listings were then read multiple times, discussed by all members of the research team, and analyzed using inductive content analysis to verify information collected during the focus group ( Potter, Gordon and Hamer, 2004; Patton, 2015). Similar ideas were grouped and checked against transcript and chat log data during both the sharing of ideas and discussion steps of the NGT process. Using an iterative approach, similar ideas were combined, and a final list of best practices was created. Quotes were extracted to explain individual and group thinking ( Potter, Gordon and Hamer, 2004).

During data analysis, we realized that 11 key ideas (two from the feedback category, and nine from collegial exchange) were not voted on due to a technical malfunction within our voting spreadsheet. A second meeting with four participant experts, and individual meetings with three remaining participant experts were conducted from December 2018 to January 2019 to finalize voting on the 11 remaining key ideas. The sessions were also conducted using Adobe Connect technology and lasted 30 minutes. First, we reintroduced the study information including definition of a best-practice and research questions. Then, participant experts silently voted on the 11 remaining ideas, and each meeting concluded with a brief presentation of our preliminary results. We considered this process a continuation of our original focus group as it included the same participant experts, and no idea generation or discussions took place.

Our research team did not have pre-existing relationships with the participant experts. Additionally, we had no previous experience developing or managing peer coaching initiatives that might have influenced our data gathering or analysis. However, we acknowledge that findings from our website review may have influenced data interpretation and analysis. An audit trail was maintained to enhance trustworthiness.

## Results/Analysis

We conducted a focus group with seven faculty developers representing peer coaching initiatives at seven medical schools across the United States. Together they generated and voted on 64 key ideas. Forty key ideas were considered best practices. Then, through data analysis, the list was consolidated into 17 best practices of peer coaching for medical educators that optimize teaching effectiveness ( Table 1). Best practices are presented within the categories of peer observation of teaching, feedback, and collegial exchange. Of note, during the discussion stage of the NGT, we observed that participant experts were eager to share experiences and findings from their own institutions, and they seemed interested in future collaboration. Additionally, we found that video-teleconference provides a feasible way to conduct a focus group using NGT. In the results and discussion that follows, the peer coach is the medical educator who is observing the teaching session, and the observed educator is the medical educator being observed.

### Best Practices:

#### Peer Observation of Teaching

For peer observation of teaching, participant experts unanimously agreed that the peer observation process must have a framework. Suggested elements of the framework included a pre-observation meeting, the direct observation, a post-observation debrief, and a self-assessment. Participant experts felt that the pre-observation meeting was useful to negotiate goals for the observation. At this meeting, the peer coach gains a better understanding of what the observed educator wants to address during the teaching observation. Simultaneously, this meeting provides peer coaches with the opportunity to explain their role during the observation process. For example, they may address that they do not participate in teaching. Peer coaches may also communicate that it is important for the learners to understand they themselves are not being evaluated by the peer coach. A separate post-observation debrief should occur soon after the observation. During the debrief, the peer coach provides feedback to the observed educator. Reflection and collegial discussions on teaching occur. Finally, the self-assessment, which could be performed before or after observation, had several uses. When undertaken before the observation, it helps the educator identify learning needs and goals for the teaching session. Alternately, self-assessment following the observation provides a formal opportunity for the educator to reflect on the teaching session.

#### Feedback

All participant experts felt oral and written feedback should remain confidential. Participant experts also indicated that peer coaches should be trained to deliver timely, specific feedback, that is behaviorally based. Examples of teaching behaviors should be provided as well as constructive suggestions for how to change the behavior. Participant experts agreed that peer coaches should be trained to deliver feedback as a dialogue, encouraging conversations on teaching. Finally, they recommended that the peer coach help the observed educator create a developmental action plan based on the feedback provided, and goals should be developed for the next teaching session.

#### Collegial Exchange

The best practices for collegial exchange described ideal environmental conditions to foster collegial exchange. Participant experts recommended that faculty developers create an environment that is perceived to be safe, respectful and non-threatening. The environment was considered both a physical and emotional space. For example, one expert felt the physical space should be private to foster collegial exchange. Emotionally, educators needed to perceive the peer coach as trustworthy, honest, credible and desiring to help. One expert said: “There has to be a culture of psychological safety..having a trusted colleague and potentially encouraging reciprocal observation to help set up that feeling.”

Participant experts also recommended creating a culture focused on improvement, growth and mastery. To accomplish this, they felt peer coaching initiatives should have clear objectives that utilize a formative assessment process to develop the educator, not evaluate them. For example, one participant remarked: “I agree with putting formative in the culture (collegial exchange). It’s what needs to occur at an institutional level to support the idea of coaching for growth and mastery.” Participant experts recommended participation be voluntary-driven by self-motivation to improve in certain areas of teaching. Additionally, participant experts felt that peer coaching should contribute to a larger professional development plan for the educator. Finally, participant experts agreed that peer coaching must be supported at all levels within an organization to foster collegial exchange and to create a cultural acceptance of peer observation.

**Table 1.** Seventeen Best Practices derived from expert consensus and written for faculty developers

	Best Practice	Selected Illustrative Quotes
**Peer Observation of Teaching**	Plan a pre-observation meeting to negotiate goals for the teaching sessionEnsure the peer coach’s role during the observation is understoodPlan a post-observation debrief to provide feedbackImplement a self-assessment strategyImplement a standardized training process for peer coaches	“I had timing of the events, um which means making sure that you have to schedule a pre-meeting, a post-meeting, and the lecture/observation time.” “The role of the observer in the learning session needs to be defined. So, they do not participate in the teaching and the learners understand they’re not being evaluated.” “To me it makes sense to have self-assessment as the fourth piece of the observation process.”
**Feedback**	Maintain confidentiality of feedbackTrain peer coaches to do the following:Provide behaviorally-based feedbackEncourage dialogueProvide specific feedbackProvide timely feedbackHelp medical educators develop a behaviorally-based action plan and define teaching goals to work toward	“It needs to be a confidential process...the forms and whatever paperwork that comes out of it only is seen by the observer and the teacher.” “It’s fact-based and not subjective. Behaviors that can be changed and giving examples of behaviors.” “I said that the person giving the feedback should facilitate a discussion through dialogue. So, kind of facilitating a conversation as opposed to spewing observations.”
**Collegial Exchange**	Create a culture that emphasizes improvement, growth and mastery of teaching skillsUtilize a formative assessment processCreate an environment that is perceived as safe, non-threatening, and respectfulMake participation voluntarySelect peer coaches who are trustworthy, honest, credible, and desire to helpGarner support for peer coaching at all organizational levels (department, medical school, university)	“...that mastery or growth-oriented mindset. Meaning there’s this idea that we all can improve.” “I consider peer observation to be, always a formative process. If it’s summative, for me that leads me into thinking that it’s going to be associated with promotion, tenure, or some other evaluation.” “Create a safe environment to elicit feedback, and so this is all non-punitive, it’s to grow the educator, and it should be made clear.” “I think it needs to be supported not just at the level of your department, but the level of either your medical school or university. So, it needs to be more of the vertical levels of support.”

### Should Have and Not Required:

While not considered best practices, the voting process also identified key ideas that participant experts considered ‘Should Have’ and ‘Not Required.’ We report these key ideas in appendices as faculty developers may find them useful, and they may generate questions for future research. The ideas deemed as ‘Should Have’ were considered a standard component of a peer coaching initiative, but they did not optimize teaching effectiveness (Appendix 1). In particular, three key ideas contributed to significant discussion amongst participants: 1) the type of training and expertise required of the peer coach, 2) the use of observation forms, and 3) whether feedback should be generalizable. The key ideas deemed as ‘Not Required’ were not standard components of an initiative, and they did not optimize teaching effectiveness (Appendix 2). Key ideas focused on details related to reciprocal observation and whether faculty developers should assign peer coaches or let educators choose their own peer coach (either from a predetermined list or from peers within their own department or section).

## Discussion

In the following sections, we draw attention to main points from the best practices within each category. Based on these best practices, we suggest several practical applications for faculty developers and highlight areas for future research.

### Peer Observation of Teaching:

The participant experts emphasized the best practice of using a framework to structure the peer observation process. The use of an observation framework is well supported in the literature. The most frequently utilized framework is a 3-phase process model that includes a pre-observation meeting to set goals for the teaching session, direct observation of teaching, and post-observation feedback ( Martin and Double, 1998). Extending this model, the participant experts recommended integrating self-assessment, which is a practice supported by the literature ( Joyce and Showers, 1982; Garcia *et al.*, 2017), to promote reflection on teaching and/or set goals for the observation of teaching session. When discussing self-assessment, the participant experts were not prescriptive in how the self-assessments should be conducted (e.g., during the pre-observation meeting, during the post-observation debrief, in writing, orally). Future researchers might consider studying timing, modalities and structures for self-assessment to determine the most efficacious approach for peer coaching.

The participant experts advocated for standardized training of peer coaches but were not directive regarding exact details to be standardized. While there is some literature on peer coach training for medical educators ( Siddiqui, Jonas-Dwyer and Carr, 2007; Newman *et al.*, 2009), it tends to focus at the individual level or in a specific domain (e.g., in simulated settings ( Cheng *et al.*, 2017)). Furthermore, it provides limited guidance for faculty developers hoping to create program-level training plans that can be replicated and validated at other institutions. Additional challenges with creating a standardized training program include the influence of external stakeholders and the unique requirements of the teaching environment (e.g., clinical versus classroom settings). Despite these challenges, we propose it is possible to create a standardized training program for peer coaches. To accomplish this, we suggest faculty developers consider creating a peer coaching learning community similar to the American Association of Medical Colleges’ (AAMC) Affinity Groups to foster collaboration, networking, and future research.

### Feedback:

Feedback was a significant area of interest for our participants. Thus, we chose to focus on two best practices of feedback that participant experts underscored: maintaining confidentiality and providing behaviorally-based feedback. Maintaining confidentiality of feedback reinforces to the observed educator that peer coaching is developmental and non-evaluative ( Gosling, 2002; Alvey and Barclay, 2007; Huston and Weaver, 2008; Cox, 2012).To maintain confidentiality, participant experts recommend conducting the post-observation debrief in a private location and securing all written feedback. Martin and Double suggest that confidentiality of feedback (both oral and written) should be discussed at the pre-observation meeting ( Martin and Double, 1998). If the peer coaching initiative requires documentation that coaching took place, observed educators must feel confident that this information will not result in an evaluation of their teaching. Huston and Weaver offer several recommendations on how to assess peer coaching initiatives while maintaining confidentiality ( Huston and Weaver, 2008). Finally, as digitization makes communication more efficient, faculty developers may look to online platforms to document participation and share feedback. We suggest considering a confidential web-based feedback system like SPARK that has proven effectiveness in other fields ( Freeman and McKenzie, 2002).

The best practice of providing behaviorally-based feedback aligns with recent recommendations for peer feedback and feedback in clinical education ( Newman, Roberts and Frankl, 2018).Participant experts recommended that faculty developers train peer coaches to focus the feedback discussion on observed teaching behaviors (preferably those identified by the observed educator during the pre-observation meeting). Newman, Roberts, and Frankl called this ‘focusing feedback on teaching skills or methods’ as opposed to feedback oriented to the person, and their article provides specific examples ( Newman, Roberts and Frankl, 2018).

### Collegial Exchange:

To encourage collegial exchange, participant experts identified several best practices (e.g., utilize a formative assessment process; encourage voluntary participation; create a safe, respectful, non-threatening environment) that foster trust within peer coaching relationships. Trust is defined as: “the willingness of a party to be vulnerable to the actions of another party based on the expectation that the other will perform a particular action important to the trustor, irrespective of the ability to monitor or control that other” ( Mayer, Davis and Schoorman, 1995). Trust has been examined in the peer coaching literature ( Eisen, 2001; Cox, 2012). For example, Eisen studied the peer learning partnerships of community college faculty and identified trust as the most essential aspect of the peer dynamic ( Eisen, 2001). In particular, the absence of hierarchical relationships, non-evaluative feedback, and voluntary participation helped equalize power relationships and contributed to trust building ( Eisen, 2001). Once trust is established within the peer coaching partnership, the observed educator is more likely to engage in collegial exchange which supports active experimentation and higher transfer of training to the teaching environment ( Showers, 1984).

Designing peer coaching initiatives as voluntary, formative assessment processes separate from peer evaluation is supported by research in higher education and medical education ( Eisen, 2001; Gosling, 2002; Adshead, White and Stephenson, 2006). Additionally, our participant experts felt that faculty developers should select peer coaches who are considered trustworthy, honest, credible, and convey a desire to help. These design elements and attributes foster trust, and establish a safe, non-threatening, respectful environment within which learning can take place ( Eisen, 2001; Edmondson, Kramer and Cook, 2004; Cox, 2012). We suggest faculty developers thoughtfully consider how their peer coaching initiative is advertised. If participation is voluntary, how are faculty motivated to participate? Does the advertisement reflect the formative nature of the initiative? Additionally, faculty developers should consider re-evaluating their peer coach selection criteria to ensure they are actively searching for the aforementioned attributes within faculty performance reports, teaching portfolios, interviews, and letters of intent.

### Limitations:

Our study was limited to only one focus group of faculty developers. Thus, we did not evaluate peer coaching initiative design and implementation from the perspectives of peer coaches, observed educators, or academic leaders. Nevertheless, the medical schools represented were geographically diverse, and this is the first study to our knowledge that promotes collaboration among experts in peer coaching for medical educators. The amount of time between focus group meetings may have altered voting patterns as the context of the idea sharing and discussion steps was missing. However, we did provide a handout and slides to remind participants of the research study design, best practice definition, and research questions. We also used the same Adobe Connect teleconference software and voting format to ensure participants were familiar with the technology and procedures. Finally, when participants required clarification of an idea, transcript material was presented to them provide context.

## Conclusion

Through expert consensus, we generated 17 best practices of peer coaching for medical educators that optimize teaching effectiveness. Our results provide a practical resource for faculty developers to utilize when developing peer coaching initiatives. In our efforts, we also demonstrated the feasibility of conducting a focus group using NGT with video-teleconference software. Moving forward, collaboration is needed to create a standardized peer coach training program that can be disseminated to multiple institutions. Additionally, exploring the perspectives of peer coaches, observed educators, and academic leaders may uncover diverging viewpoints that influence initiative design and implementation.

## Take Home Messages

• Through expert consensus, we generated 17 best practices of peer coaching that can be applied by faculty developers to create effective initiatives.
• The peer coaching process is supported by an observation framework that includes a pre-observation meeting, an observation, a post-observation debrief, and a self-assessment.
• Maintaining confidentiality of feedback, making participation voluntary, and utilizing a formative assessment process is important.
• Conducting a focus group using NGT is feasible with video-teleconference software.
• Future research should be collaborative and should seek to uncover divergent perspectives on initiative design and implementation.

## Notes On Contributors

Adriane E. Bell is a Master of Health Professions Education candidate and an assistant professor of Family Medicine within the Department of Family Medicine, F. Edward Hébert School of Medicine, Uniformed Services University of the Health Sciences in Bethesda, Maryland, USA. https://orcid.org/0000-0001-6455-0835

Holly S. Meyer is the assistant director of Student Affairs and an assistant professor of Medicine in the Division of Health Professions Education within the Department of Medicine, F. Edward Hébert School of Medicine, Uniformed Services University of the Health Sciences in Bethesda, Maryland, USA. https://orcid.org/0000-0001-8833-8003

Lauren A. Maggio is the associate director of Distributed Learning and Technology and associate professor of Medicine in the Division of Health Professions Education within the Department of Medicine, F. Edward Hébert School of Medicine, Uniformed Services University of the Health Sciences in Bethesda, Maryland, USA. https://orcid.org/0000-0002-2997-6133

**Disclaimer:** The views expressed in this article are those of the authors and do not necessarily reflect the official policy or position of the Uniformed Services University of the Health Sciences, the Department of Defense, or the U.S. Government.

Written work prepared by employees of the Federal Government as part of their official duties is, under the U.S. Copyright Act, a “work of the United States Government” for which copyright protection under Title 17 of the United States Code is not available. As such, copyright does not extend to the contributions of employees of the Federal Government.

## Appendices

**Appendix 1.** Key ideas that were voted as ‘Should Have’ by the participant experts

	Key Ideas	Selected Illustrative Quotes
**Peer Observation of Teaching**	Help educators develop a reflective practiceSelect peer coaches who are effective educatorsUse a form to set goals for the teaching session and to frame the self-assessmentUse a structured form to focus the observation and generate feedback (it can be tailored to the teaching environment)	“I think forms are really helpful so that the observers have some type of shared mental model around what it is that are important or effective behaviors to be paying attention to.” “We have forms that are for the classroom, we have forms that are for basic sciences, we have forms that are for the proceduralist...The reasons why we have varied forms is because we don’t have content experts coming in to do the observation, and it’s just helpful for those who don’t fully understand that environment.”
**Feedback**	Begin by eliciting the ‘frame’ of educator using the Feedback with Good Judgement Model Provide complimentary feedbackMake feedback generalizableSelect peer coaches with content knowledgeProvide the peer coach with knowledge and access to resources beyond their expertise to foster good conversations and promote effective feedback	Referencing Feedback with Good Judgement, “Why did you do it this way?” “Ah it should be complementary and focused on the things the faculty member did well. As well as evoking a sense of “we” as opposed to “you.”” “The idea that it’s not a single session with one observer as the whole basis for feedback.” “I think that content expertise matters when there are very advanced learners.” “It would be most helpful I think if the person observing has an idea of resources that are available, so that not only are there specific behavior changes being recommended, but perhaps resources that can be tapped into within that environment.”
**Collegial Exchange**	Include educators at multiple skill levelsCreate a culture to adopt peer observation	“And I think it’s really important that it include educators at multiple skill levels...I think it’s important to have a culture to recognize where everybody can improve, and it’s not just those who may be less experienced or in the midpoint in their career, but even those who are more senior can always do better in terms of their teaching.” “Thinking about how you foster an environment where everybody is open to the peer observation process.”

**Appendix 2.** Key ideas that were voted as ‘Not Required’ by the participant experts

	Key Ideas	Selected Illustrative Quotes
**Peer Observation of Teaching**	Help the peer coach gain skills through the peer observation processSchedule a follow-up observation	“... that the observer should gain skills in the process. In other words, it’s not only driven on improving the skills of the person being observed.” “And also, also there should be a follow-up observation. So, after the, the pre-meeting, the initial observation, there should be a follow-up observation as well”
**Collegial Exchange**	Require all teaching faculty to participate in peer coaching--observing and being observed teachingCreate peer coaching pairs within departments/programsPromote a sense of equality between the peer coach and educator (nonhierarchical)Establish ongoing relationships between the peer coach and observed educatorHave the peer coach and observed educator watch a recording of teaching togetherAssign the peer coachAllow the observed educator to choose the peer coach	“We feel that observing and being observed is, is a key part of being a reflective teacher.” “Another way for fostering collegial exchange is for departments or programs to set up peer observation coaching pairs within the department.” Referring to the environment, “shows a sense of equality between the observer and observee, so not hierarchical.” “Sometimes we’ll use recordings, where actually teaching is being recorded and having an infrastructure for that, so that pairs can actually watch the teaching.” “At our institution we have Academy members...and so we are training them as our initial peer observers, and that could be the list that we share with our faculty to choose from.”

*No key ideas related to feedback were voted as ‘Not Required’ by the participant experts

## Declarations

The author has declared that there are no conflicts of interest.

## Ethics Statement

The Uniformed Services University of the Health Sciences (USU) institutional review board declared this study exempt from further review (Protocol Number: T0839444).

## External Funding

This article has not had any External Funding
