# Supplementary material for: On the Same Page: Building Best Practices of Peer Coaching for Medical Educators Using Nominal Group Technique
Source: MedEdPublish (2016). 2019 Apr 25;8:95. [Version 1] doi: 10.15694/mep.2019.000095.1 (PMC10712581; doi:10.15694/mep.2019.000095.1)
Supplement: Peer review history

# Reviewer report: Reviewer response for version 1

**DOI:** 10.21956/mep.19837.r29911

**Published:** 2019-05-08

**Authors:** Trevor Gibbs

**Affiliations:** 1 AMEE

This review has been migrated. The reviewer awarded 4 stars out of 5 As we have an even greater problem with balancing teaching, research and service activities, then papers that discuss peer and near-peer activities become more important. I was a little confused as to whether this paper was about the nominal group technique or the discussion of the 17 best practice points for peer coaching. Whatever, I enjoyed reading the paper and learned from it. A good read for those involved in curriculum design and resource allocation.

Reviewer Expertise:

NA

No decision status is available

# Reviewer report: Reviewer response for version 1

**DOI:** 10.21956/mep.19837.r29909

**Published:** 2019-05-02

**Authors:** Susan Van Schalkwyk

**Affiliations:** 1 Stellenbosch University

This review has been migrated. The reviewer awarded 4 stars out of 5 I was attracted to this article by the title, both in terms of the idea of peer coaching for medical educators as well as the use of a nominal group technique (NGT). I agree with the authors’ claim that this sort of work can provide those responsible for faculty development initiatives with tools and frameworks that can enhance their practice. The article provides a sound overview of some of the most current literature around peer coaching, acknowledging the lack of consensus with regard to approaches most likely to optimise ‘teaching effectiveness’. I was particularly interested in the use of NGT online and would have liked to have read more about any challenges that emerged from using this approach, even potential pitfalls that others should look out for. The FGs were centred around three components of peer coaching as inferred from the literature. I was, however, curious as to whether best practices were mentioned that fell outside these three components and whether offering the participants the opportunity to reflect beyond these three might not have opened up new insights – perhaps something to consider in a future study? The findings from the study resonated with what is known to foster professional learning generally – the value of peer observation, the need for some sort of structure, the emphasis on confidentiality, the role of feedback, and the value of collegial exchange based on relationships of trust, and so forth, are not new ideas. However, collating the findings into a set of best practices provides a useful framework, particularly for those starting out with such a peer coaching initiative. The authors acknowledge the small scale of the study as being a limitation, and I agree that further work in this area would be of relevance.

Reviewer Expertise:

NA

No decision status is available

# Reviewer report: Reviewer response for version 1

**DOI:** 10.21956/mep.19837.r29910

**Published:** 2019-05-02

**Authors:** BALAJI ARUMUGAM

**Affiliations:** 1 TAGORE MEDICAL COLLEGE AND HOSPITAL

This review has been migrated. The reviewer awarded 4 stars out of 5 Thanks for the invite. This article mainly speaks about the best practices for peer coaching for medical educators using novel qualitative method of approach called Nominal group technique which is commonly used for validation process. I personally appreciate the entire team for the methodology adapted to explore the best practices and finally reaching a consensus of 17 practices. Conducting the focus group discussion through video conferencing and voting finally on deciding the best practices was well planned and executed. The article was well written and clearly expressed the messages discussed in the NGT. So as a reviewer to conclude, as the illustrative quotes in best practice table says that “I think it needs to be supported not just at the level of the department, but the level of either your medical school or university. So, it needs to be more of the vertical levels of support, is needed to implement the practices of peer coaching for medical educators.

Reviewer Expertise:

NA

No decision status is available

# Reviewer report: Reviewer response for version 1

**DOI:** 10.21956/mep.19837.r29912

**Published:** 2019-04-25

**Authors:** Felix Silwimba

**Affiliations:** 1 University of Lusaka

This review has been migrated. The reviewer awarded 5 stars out of 5 The article Peer counselling for medical educators, is clear, well explained and easy to follow. The methods of using nominal group technique is fine and easily reproducible everywhere. Perhaps with a variation in use of technology. Agreeably, its application in medical schools with contribute to the quality of medical education through standardization and collaboration among medical educators within the institution and across medical training facilities.

Reviewer Expertise:

NA

No decision status is available
